# Accurate Pathological Prediction of Small Breast Cancer With Pathological Component-based Image Evaluation: A Case Report

**DOI:** 10.7759/cureus.78026

**Published:** 2025-01-26

**Authors:** Yurie Kitano, Shoji Oura, Mariko Honda

**Affiliations:** 1 Department of Surgery, Kishiwada Tokushukai Hospital, Kishiwada City, JPN; 2 Department of Surgery, Izumiotsu Municipal Hospital, Izumiotsu City, JPN

**Keywords:** breast cancer, fat, fibrous component, image evaluation, tumor cell

## Abstract

Routine mammography screening in a 63-year-old woman showed a small breast mass with indistinct borders. Ultrasound showed a triangle mass, 12 mm in size, which had somewhat unclear anterior borders with focal strong high echoes at their center, multiple punctate echogenic foci in the internal low echoes, and slightly attenuated posterior echoes. Magnetic resonance images (MRI) of the tumor showed a hypointense pattern both on T1- and fat-suppressed T2-weighted images. Time-signal intensity images of MRI showed weak early enhancement followed by a persistent pattern. These imaging findings suggested that cancer cells with some kind of papillary structures would be present sparsely and diffusely in the fibrous background. After the pathological confirmation of malignant cells, the patient underwent a partial mastectomy and sentinel node biopsy. Postoperative pathological study showed that atypical cells growing in a tubular fashion spread sparsely and diffusely in the fibrous background extending to the tumor edges. In conclusion, by understanding how collagen fibers, fat, and cancer cells affect image depiction in each imaging modality, diagnostic physicians can accurately predict the pathological findings even of small breast cancer.

## Introduction

Mammography (MMG) is a comprehensive breast imaging technique that can show the entire breast tissue and, therefore, has been the mainstay in the image evaluation of breast cancer for decades. Mammographic density is determined by the X-ray attenuation coefficient of the tissue. Therefore, depending on their X-ray attenuation coefficients, breast tumors and fat appear whitish and blackish on MMG, respectively.

Ultrasound (USG) can show tomographic planes and reveal small breast tumors even when they are located in the dense breast [1]. Reflection and backscattering of USG waves determine the shape and echogenicity of the tumor, respectively [2,3]. Internal echoes become higher as USG backscattering increases. Conversely, less USG wave backscattering makes the internal echoes of the tumor low.

Magnetic resonance imaging (MRI) helps in judging whether the target lesion is malignant or benign, especially by time-signal intensity patterns [4]. In addition, MRI evaluation is frequently done after the diagnosis of breast cancer to detect the presence of multiple lesions and intraductal spread for deciding the optimal surgical option [5]. Moreover, MRI can also be used to predict the presence of various pathological components such as collagen fibers, fat, and tumor cells through the different image-forming mechanisms besides MMG and USG.

We herein report a case of small breast cancer in which its pathologic findings were predicted not by pattern recognition but by pathological component-based image evaluation.

## Case presentation

**Figure 1:**
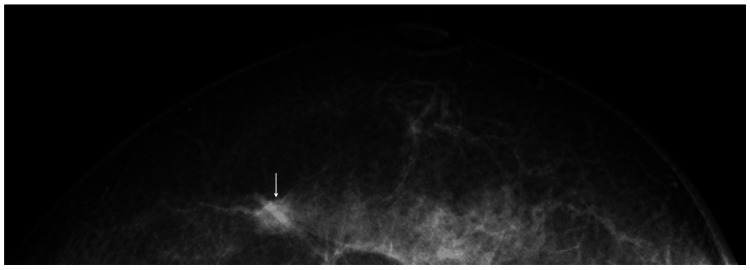
Mammography showing a dense mass with poorly defined borders in the fatty breast.

A 63-year-old woman had come to the hospital for a regular screening mammography, which showed a small mass with indistinct borders in the upper outer quadrant of her left breast (Figure 1).

**Figure 2:**
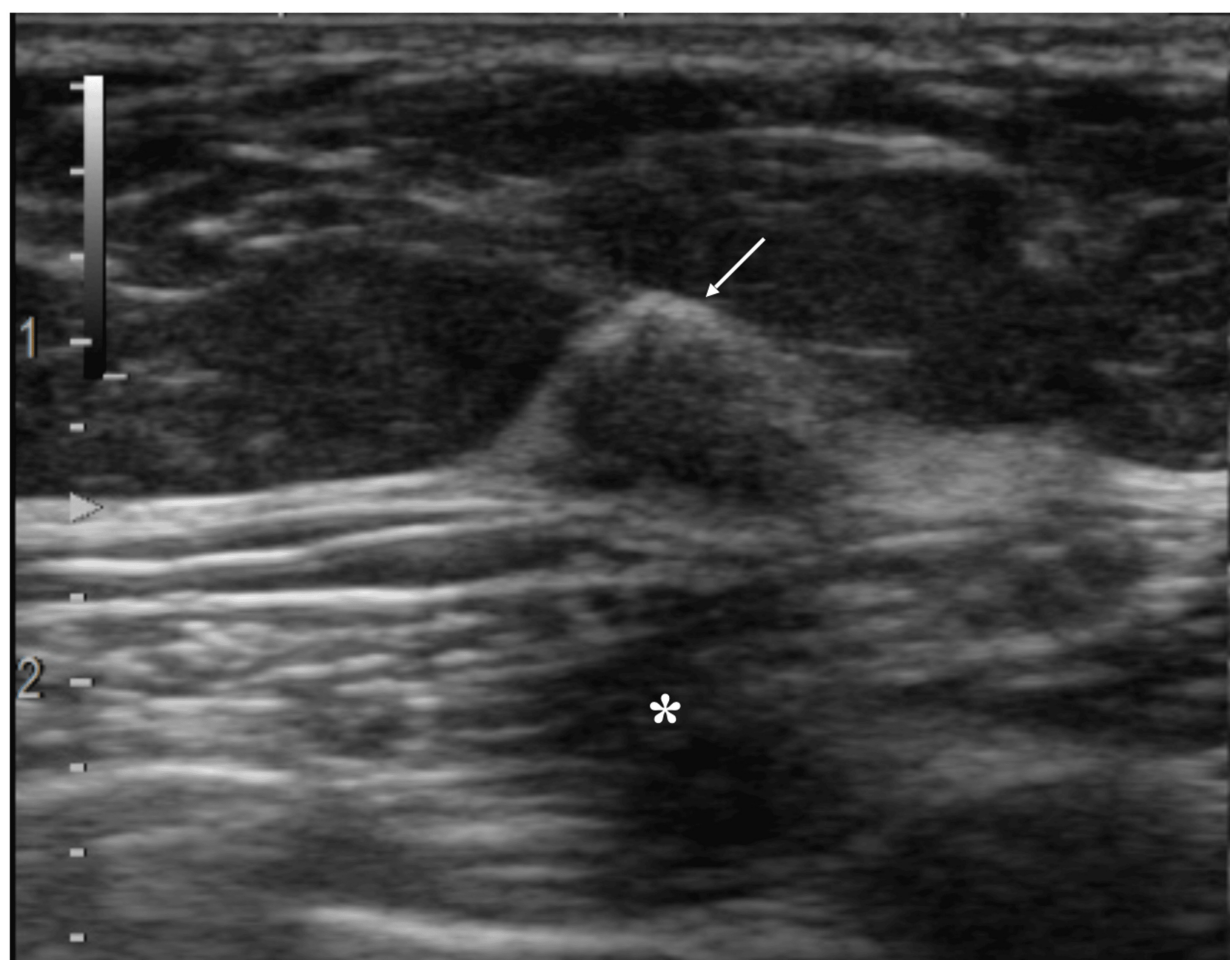
Ultrasound showing a triangular mass with somewhat unclear borders; central part of the anterior borders had very high echogenicity (arrow), while the lateral parts had less echogenicity. The mass had multiple punctate echogenic foci and slightly attenuated posterior echoes (asterisk), strongly suggesting some kind of papillary structures and widespread collagen fibers in the tumor.

A USG was done, which showed a triangular mass of 12 mm in size with somewhat unclear anterior borders with focal strong echoes at their center, multiple punctate echogenic foci in the internal low echoes, and slightly attenuated posterior echoes (Figure 2).

**Figure 3:**
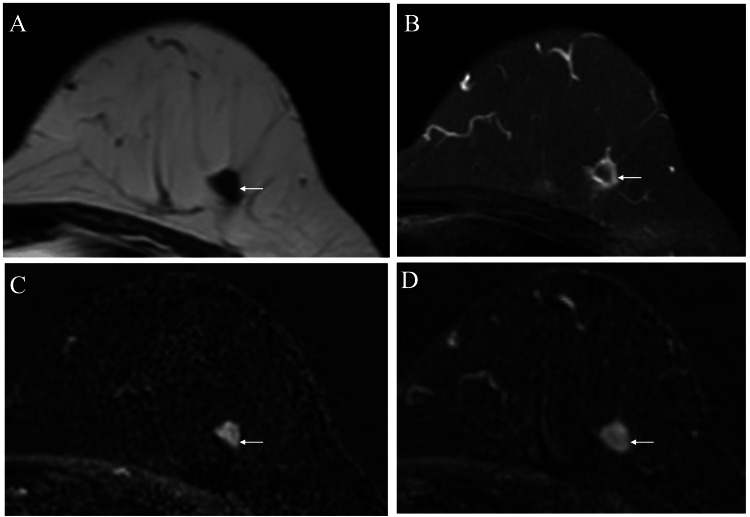
Magnetic resonance imaging (MRI) findings of the tumor (A) MRI of the tumor (arrow) showing a hypointense pattern on T1-weighted images. (B) MRI showing a hypointense mass encircled by a hyperintense rim (arrow) on fat-suppressed T2-weighted images. (C) Time-signal intensity image showing a weak enhancement (arrow) on early arterial phase images. (D) Time-signal intensity image showing a retained weak enhancement (arrow) on delayed phase images.

MRI of the tumor showed a hypointense pattern both on T1- and fat-suppressed T2-weighted images (Figures 3A, 3B). In addition, the tumor was encompassed by a hyperintense rim on fat-suppressed T2-weighted images. Time-signal intensity images of the tumor showed weak early enhancement and a persistent pattern (Figures 3C, 3D).

All these imaging findings suggested that cancer cells with some kind of papillary/tubular structures would be present sparsely and diffusely in the fibrous background. Core needle biopsy of the tumor pathologically showed atypical cells growing in tubular and cord-like fashions with a proliferation of collagen fibers, leading to the diagnosis of scirrhous type invasive ductal carcinoma. Therefore, with a preoperative diagnosis of node-negative breast cancer, the patient underwent partial mastectomy and sentinel node biopsy.

**Figure 4:**
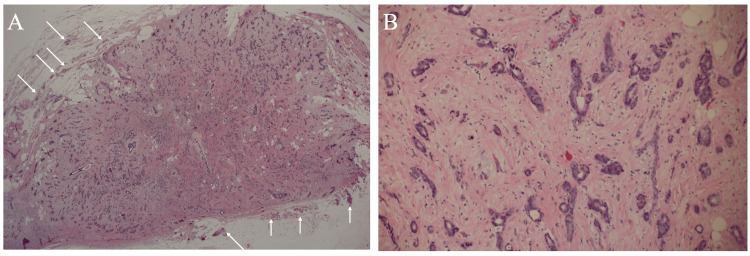
Pathological findings A. Low-magnified view (H&E ×20) showing tumor cells sparsely distributed throughout the mass in the fibrous background. Tumor cells and micro-vessels (arrows) are present around the tumor without any accompanying fibrous components, which presumably contributed to generate the hyperintense rim on fat-suppressed T2-weighted images. B. Magnified view (H&E ×200) showing tumor cells, mainly growing a tubular fashion, proliferated sparsely in the tumor against the background of collagen fibers.

The bisected cut surface macroscopically showed a well-demarcated tumor encompassed by fat tissue. Pathologically, atypical cells were growing mainly in a tubular fashion and distributed sparsely and diffusely in the fibrous background extending widely throughout the tumor (Figure 4), matching well with our prediction of pathological findings.

Immunostaining showed that the tumor cells were highly positive for estrogen receptors, were negative both for progesterone receptors and human epidermal growth factor receptor type 2, and had a Ki-67 labeling index of 6%. The patient received adjuvant radiotherapy to the conserved breast and has been well for 18 months on letrozole therapy.

## Discussion

Collagen fibers have fewer protons and a higher USG attenuation coefficient than other pathological components in the breast [6]. MRI uses the magnetic properties of protons to generate images. Fewer protons, therefore, caused the hypointense pattern of the tumor on fat-suppressed T2-weighted images and the persistent pattern on time-signal intensity images, respectively. The high USG attenuation coefficient generally makes the posterior echoes attenuated. However, the small tumor size of 12 mm only generated slight USG attenuation in this case. In addition, collagen fibers, when present at the tumor edges mixed with tumor cells, obscure the tumor borders. The tumor borders, therefore, were indistinct both on USG and MMG in this case.

Fat has a very small X-ray attenuation coefficient, the lowest acoustic impedance in the body, and markedly different T1- and T2-values than mammary gland and breast tumors [7-10]. The very small X-ray attenuation coefficient of the fat surrounding the tumor made the small tumor stand out on MMG. The lowest acoustic impedance of fat generated strong USG wave reflections at the plane in contact with the tumor [7,9,10] and actually made the center of the anterior tumor borders highly echogenic in this case. However, the outer part of the anterior tumor borders had a decreased echogenicity, which was presumably caused by less USG wave reflection to the USG probe due to the inclination of anterior tumor borders. Markedly different T1- and T2-values of fat made the tumor depiction very clear both on T1- and fat-suppressed T2-weighted images [11]. The hypointense pattern on fat-suppressed T2-weighted images suggested the massive presence of fat or collagen fibers in the tumor. T1-weighted images, however, would have shown a more hyperintense pattern if massive fat had been present in the tumor. MRI findings, therefore, highly suggested the abundant presence of collagen fibers in the tumor. In addition, rim enhancement on fat-suppressed T2-weighted images was likely due to the presence of small vessels surrounding the mass.

Tumor cells have high X-ray attenuation coefficients and abundant protons. Diffuse and sparse cancer cell distribution in the fibrous background well explained the depiction of the somewhat hyper-dense mass with indistinct borders on mammography, the hypointense pattern on fat-suppressed T2-weighted images, and the plateau pattern on time-signal intensity images. In addition, tumor cells, when forming some kind of papillary/tubular structures, make the internal echoes high due to the papillary/tubular structure-induced USG wave backscattering [12]. Therefore, the diffuse distribution of tumor cells with a tubular pattern generated the multiple punctate echogenic foci in this case.

The vast majority of physicians generally diagnose various solid tumors with pattern recognition of image findings. In short, many physicians understand that scirrhous-type invasive ductal carcinomas typically have an irregular shape, low internal echoes, attenuated posterior echoes, and large depth/width ratios on USG. Diagnostic physicians, however, often are unable to make a correct diagnosis through this approach, especially in the diagnosis of small breast cancers. It is, therefore, very important to understand how fibrous components, fat, and tumor cells affect the image depiction in each image modality.

## Conclusions

Fibrous components obscure the tumor borders when present at the tumor edges mixed with cancer cells, make the posterior echoes attenuated, depict the tumor as hypointense on T2-weighted images, and show a plateau or persistent pattern on time-signal intensity images. Fat appears blackish on MMG due to its low X-ray attenuation coefficient, highlights adjacent tumors or materials on ultrasound due to its extremely low acoustic impedance, generates a hyperintense pattern on both T1- and T2-weighted images, and has a plateau or persistent pattern on time-signal intensity images. Tumor cells appear whitish on MMG due to their high X-ray attenuation coefficients, appear blackish and whitish when showing solid growth and when having papillary structures, respectively, on USG, appear hyperintense on T2-weighted images due to the abundance of protons, and show a washout pattern when the density of tumor cells is high on time-signal intensity images. Therefore, these pathological component-based image evaluations can help us accurately predict the pathological findings even of small breast cancer.
